# Complete Genome Sequences of HonestAbe, Anthony, and Taffo16, Three Cluster C Bacillus cereus Group Bacteriophages

**DOI:** 10.1128/genomeA.00493-18

**Published:** 2018-06-21

**Authors:** Martin Lee, Kayla M. Puglisi, UMBC STEM-BUILD Cohort 1, Ivan Erill, Steven M. Caruso

**Affiliations:** a Department of Biological Sciences, University of Maryland, Baltimore County, Baltimore, Maryland, USA

## Abstract

Three cluster C Myoviridae bacteriophages that infect Bacillus cereus group bacteria were isolated from soil collected in the mid-Atlantic region using B. thuringiensis subsp. kurstaki as a host. Bacillus phages HonestAbe, Anthony, and Taffo16 each shared 90% or higher average nucleotide identities within their subclusters.

## GENOME ANNOUNCEMENT

Interest in Bacillus cereus group bacteria stems from their ubiquity, complications in species identification when examined phenotypically and genetically (1), and the medical relevance of several members of the group, including B. cereus *sensu stricto*, an organism responsible for outbreaks of food poisoning, B. anthracis, the causative agent of anthrax, and the agriculturally important entomopathogen B. thuringiensis (2–4). Here, we present the genome sequences of three Myoviridae bacteriophages, Bacillus phages HonestAbe, Anthony, and Taffo16, isolated from soil samples collected from sites in Maryland and Washington, DC, using B. thuringiensis subsp. kurstaki strain ATCC 33679 as a host (Table 1). This work was completed as part of the SEA-PHAGES (5) and UMBC STEM-BUILD (6) programs.

**TABLE 1:** Properties of three Bacillus phages

Phage name	Genome size (bp)	Terminal repeat size (bp)	GC content (%)	No. of open reading frames	No. of RNAs	Origin	Cluster	GenBank accession no.
Anthony	159,983	2,422	38.0	286	7	Frederick, MD, USA	C2	MF498901
HonestAbe	161,129	2,576	38.7	289	3	Washington, DC, USA	C1	MG763894
Taffo16	164,241	2,249	37.8	284	0	Columbia, MD, USA	C3	MF765814

Sequencing was performed by the Pittsburgh Bacteriophage Institute and the NC State Genomic Sciences Laboratory to approximately 100× coverage with the Illumina MiSeq platform, and the assembly was completed with Newbler and Consed (7). Genome annotation was completed using DNA Master (http://cobamide2.bio.pitt.edu). Bacillus phages HonestAbe, Anthony, and Taffo16 all have linear double-stranded DNA genomes that end in direct terminal repeats averaging 2,416 bp (standard deviation [SD], ±164 bp), as determined by examination of Illumina sequencing reads. The mean genome length was 161,784 bp (SD, ±2,203 bp), and the GC content was in the 37.8% to 38.7% range (Table 1). After removal of duplicates in the terminal repeats, the phage genomes had between 284 and 289 protein-coding genes, with an average start codon breakdown of 87% AUG, 6% GUG, and 8% UUG. Bacillus phage Anthony encodes seven tRNAs, and HonestAbe encodes three tRNAs; no tRNA genes were identified in the Taffo16 genome (Table 1).

Cluster assignment was based on nucleotide conservation, genomic synteny, and phylogenetic analysis, as described previously (8). Average nucleotide identity (ANI) was determined by OrthoANI (9). HonestAbe shares a mean of 89.7% (SD, ±3.7%) ANI with the other members of cluster C1 (*n =* 33). Anthony shares a mean of 96.6% (SD, ±1.0%) ANI with C2 phages (*n =* 10), and Taffo16 shares a mean of 92.8% (SD, ±5.9%) ANI with C3 phages (*n = 15*). See http://bacillus.phagesdb.org for details on members of clusters C1, C2, and C3.

Transmission electron microscopy showed HonestAbe, Anthony, and Taffo16 to have 158-nm, 219-nm, and 240-nm tails, as well as icosohedral capsids with diameters of 80 nm, 105 nm, and 117 nm, respectively. Both Anthony and Taffo16 were able to infect the nonpathogenic B. thuringiensis strain DSM 350 (10) as efficiently as the original host, as well as B. cereus strains FDA5 (ATCC 10702) and Gibson 971, but only at multiplicities of infection of 100- to 1,000-fold higher. Neither of them was able to infect or lyse B. anthracis delta Sterne.

### Accession number(s).

The GenBank accession numbers for the genome sequences reported here are provided in Table 1.

## References

[1] Okinaka RT, Keim P 2016 The phylogeny of *Bacillus cereus sensu lato*, p 239–251. *In* Driks A, Eichenberger P (ed), The bacterial spore: from molecules to systems. ASM Press, Washington, DC. doi:10.1128/microbiolspec.TBS-0012-2012.

[2] Schmid D, Rademacher C, Kanitz EE, Frenzel E, Simons E, Allerberger F, Ehling-Schulz M 2016 Elucidation of enterotoxigenic *Bacillus cereus* outbreaks in Austria by complementary epidemiological and microbiological investigations, 2013. Int J Food Microbiol 232:80–86. doi:10.1016/j.ijfoodmicro.2016.05.011. 27257745

[3] Beyer W, Turnbull PCB 2009 Anthrax in animals. Mol Aspects Med 30:481–489. doi:10.1016/j.mam.2009.08.004. 19723532

[4] Zheng J, Gao Q, Liu L, Liu H, Wang Y, Peng D, Ruan L, Raymond B, Sun M 2017 Comparative genomics of *Bacillus thuringiensis* reveals a path to specialized exploitation of multiple invertebrate hosts. mBio 8:e00822-17. doi:10.1128/mBio.00822-17. 28790205 PMC5550751

[5] Jordan TC, Burnett SH, Carson S, Caruso SM, Clase K, DeJong RJ, Dennehy JJ, Denver DR, Dunbar D, Elgin SCR, Findley AM, Gissendanner CR, Golebiewska UP, Guild N, Hartzog GA, Grillo WH, Hollowell GP, Hughes LE, Johnson A, King RA, Lewis LO, Li W, Rosenzweig F, Rubin MR, Saha MS, Sandoz J, Shaffer CD, Taylor B, Temple L, Vazquez E, Ware VC, Barker LP, Bradley KW, Jacobs-Sera D, Pope WH, Russell DA, Cresawn SG, Lopatto D, Bailey CP, Hatfull GF 2014 A broadly implementable research course in phage discovery and genomics for first-year undergraduate students. mBio 5:e01051-13. doi:10.1128/mBio.01051-13. 24496795 PMC3950523

[6] LaCourse WR, Sutphin KL, Ott LE, Maton KI, McDermott P, Bieberich C, Farabaugh P, Rous P 2017 Think 500, not 50! A scalable approach to student success in STEM. BMC Proc 11:24. doi:10.1186/s12919-017-0094-5. 29375665 PMC5773890

[7] Gordon D, Abajian C, Green P 1998 Consed: a graphical tool for sequence finishing. Genome Res 8:195–202. doi:10.1101/gr.8.3.195. 9521923

[8] Sauder AB, Quinn MR, Brouillette A, Caruso S, Cresawn S, Erill I, Lewis L, Loesser-Casey K, Pate M, Scott C, Stockwell S, Temple L 2016 Genomic characterization and comparison of seven *Myoviridae* bacteriophage infecting *Bacillus thuringiensis*. Virology 489:243–251. doi:10.1016/j.virol.2015.12.012. 26773385

[9] Lee I, Ouk Kim Y, Park S-C, Chun J 2016 OrthoANI: an improved algorithm and software for calculating average nucleotide identity. Int J Syst Evol Microbiol 66:1100–1103. doi:10.1099/ijsem.0.000760. 26585518

[10] Schulenburg H, Müller S 2004 Natural variation in the response of *Caenorhabditis elegans* towards *Bacillus thuringiensis*. Parasitology 128:433–443. doi:10.1017/S003118200300461X. 15151149

